# Stretchable Metamaterial Absorber Using Liquid Metal-Filled Polydimethylsiloxane (PDMS)

**DOI:** 10.3390/s16040521

**Published:** 2016-04-11

**Authors:** Kyeongseob Kim, Dongju Lee, Seunghyun Eom, Sungjoon Lim

**Affiliations:** School of Electrical and Electronics Engineering, Chung-Ang University, Seoul 156-756, Korea; kks6695@naver.com (K.K.); dongju0721@daum.net (D.L.); umsh0303@gmail.com (S.E.)

**Keywords:** liquid metal, metamaterial absorber, microfluidics, strain sensor, stretchable

## Abstract

A stretchable metamaterial absorber is proposed in this study. The stretchability was achieved by liquid metal and polydimethylsiloxane (PDMS). To inject liquid metal, microfluidic channels were fabricated using PDMS powers and microfluidic-channel frames, which were built using a three-dimensional printer. A top conductive pattern and ground plane were designed after considering the easy injection of liquid metal. The proposed metamaterial absorber comprises three layers of PDMS substrate. The top layer is for the top conductive pattern, and the bottom layer is for the meandered ground plane. Flat PDMS layers were inserted between the top and bottom PDMS layers. The measured absorptivity of the fabricated absorber was 97.8% at 18.5 GHz, and the absorption frequency increased from 18.5 to 18.65 GHz as the absorber was stretched from its original length (5.2 cm) to 6.4 cm.

## 1. Introduction

Since the first theoretical study of metamaterials by Veselago *et al.* in 1968, metamaterials have received substantial attention because of their extraordinary characteristics such as the cloaking capacity and negative indices of refraction [1]. The permeability and permittivity of metamaterials can be manipulated by using conductive patterns. Metamaterials have been studied for use in various applications such as antennas [2], sensors [3], and perfect absorbers [4,5,6,7,8,9,10,11,12,13,14,15,16]. A metamaterial absorber uses an array of unit cells, typically consisting of a top pattern and a bottom ground plane. The unit cell of the metamaterial absorber can be regarded as an electric LC (ELC) resonator. The reflection of the absorber can be eliminated by matching the impedance of the metamaterial to that of air.

Since the introduction of metamaterial absorbers by Landy *et al.* [4], research and development activities have taken place for various metamaterial absorbers, considering broadband [5,6,7,8,9,10] and narrowband [4] applications. In addition, metamaterial absorbers have been used as plasmonic sensors [11,12] and bolometers [13]. Many recent studies examined flexible and stretchable metamaterial absorbers [14,15,16]. In addition, the frequency tunability of metamaterial absorbers can be used for sensor applications [17,18]. Most tunable absorbers have been implemented by incorporating electronic tuning components such as varactor diodes [19]. Switchable absorbers were realized by connecting PIN diodes to unit cells [20,21]. Recently, microfluidic technology has been applied to a frequency-tunable metamaterial absorber [22]. The resonant frequency was tuned by changing the dielectric constant of the liquids in the microfluidic channel.

In this paper, a stretchable metamaterial absorber using liquid metal and polydimethylsiloxane (PDMS) is proposed. The metamaterial absorber is based on a PDMS substrate, which allows it to stretch. PDMS is a silicone elastomer with a Young’s modulus of less than 2 MPa, and it has recently been used as a supporting substrate for copper in flexible applications [23]. PDMS is used routinely in the preparation of microfluidic channels [24]. It is highly elastic and has a low modulus and surface energy, exhibiting good surface conformance [25]. To fabricate a stretchable absorber, liquid metal and microfluidic technology were adopted. Top conductive patterns and ground planes were obtained by injecting eutectic gallium indium (EGaIn) liquid metal into microfluidic channels. Wide meander lines were employed for a bottom ground plane to facilitate easy injection of the liquid metal. Microfluidic technologies were applied to use small quantities of samples and reagents and to perform analyte detections with a high sensitivity, low cost, and short analysis time [26]. In addition, EGaIn is low in toxicity and viscosity and it can be quickly injected into microfluidic channels. The liquid forms a solid-like oxide surface skin, improving the mechanical stability of the substance. The performance of the proposed absorber was examined by a full-wave simulation and measurements. The absorption frequency was changed by stretching the proposed absorber while maintaining a high absorptivity.

## 2. Design

A perfect absorber corresponds to having the reflection coefficient (*Г*) and transmission coefficient (*T*) set to zero. A reflection coefficient of zero can be achieved when the impedance of the absorber (*Z_M_*) is set to 377 Ω, which is the impedance of free space (*Z_0_*). Moreover, the effective permittivity and permeability of a metamaterial can be artificially tailored. When the effective permittivity (*ε_M_*) equals the effective permeability (*μ_M_*), the reflection coefficient, defined as follows, is zero:

(1) $$\Gamma \left(\omega \right)=\frac{Z_{0}-Z_{M}\left(\omega \right)}{Z_{0}+Z_{M}\left(\omega \right)}=\frac{\sqrt{\frac{\mu_{0}}{\varepsilon_{0}}}-\sqrt{\frac{\mu_{M}\left(\omega \right)}{\varepsilon_{M}\left(\omega \right)}}\sqrt{\frac{\mu_{0}}{\varepsilon_{0}}}}{\sqrt{\frac{\mu_{0}}{\varepsilon_{0}}}+\sqrt{\frac{\mu_{M}\left(\omega \right)}{\varepsilon_{M}\left(\omega \right)}}\sqrt{\frac{\mu_{0}}{\varepsilon_{0}}}}$$

where *ε*_0_, *μ*_0_, *ε_M_*, and *μ_M_* are the permittivity of free space, permeability of free space, effective permittivity, and effective permeability, respectively. In addition to a reflection coefficient of zero, a transmission coefficient of zero can be achieved through dielectric losses of the metamaterial. Therefore, the metamaterial absorber was designed with a periodic array of ELC resonators. For a dispersive metamaterial, the resonant frequency is given as

(2) $$f=\frac{1}{2\pi \sqrt{\varepsilon_{M}\mu_{M}}}$$

**Figure 1:**
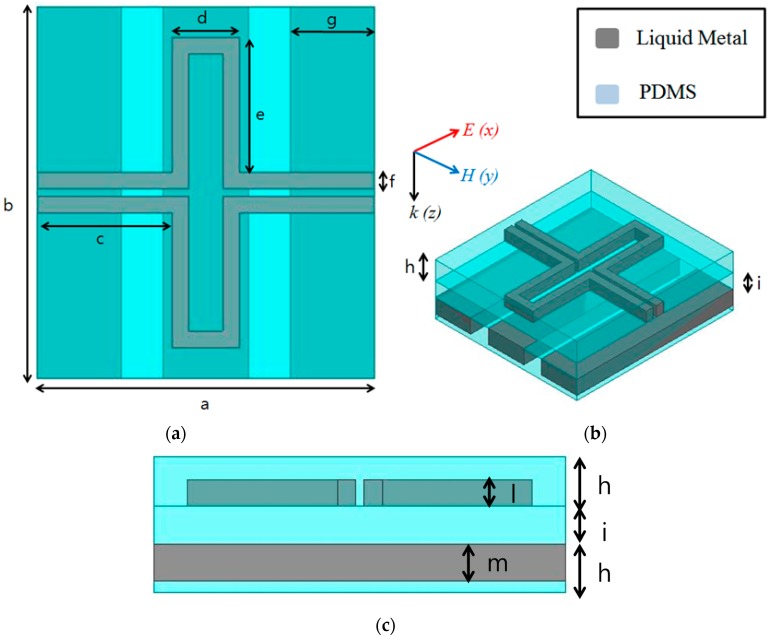
Layout of the proposed unit cells: a = 10 mm, b = 11 mm, c = 4 mm, d = 2 mm, e = 4 mm, f = 0.5 mm, g = 2.5 mm, h = 1.3 mm, i = 1 mm, l = 0.7 mm, m = 1 mm. (**a**) Top view; (**b**) bird’s-eye view; (**c**) side view.

Figure 1 illustrates the layout of the proposed unit cell for generating LC resonance. The unit cell consists of a top conductive pattern and a bottom ground plane. The LC resonance is generated from the inductance of the long conductive line as well as from the capacitance of the gap between the upper and lower conductive lines on the top pattern. There is no transmission because of the ground plane. The transmitted electromagnetic (EM) wave disappears because of dielectric losses, which can be controlled by manipulating the substrate thickness.

To make the proposed metamaterial absorber stretchable, PDMS was used as the substrate, and EGaIn liquid metal was used as the conductor. The substrate consists of three PDMS layers, as shown in Figure 1b,c. The top and bottom PDMS layers (h) are each 1.3 mm thick, and the middle PDMS layer (i) is 1.0 mm thick. To inject EGaIn, microfluidic channels were constructed on the top and bottom PDMS substrates. The middle PDMS substrate is plain, without any microfluidic channels. Therefore, the top and bottom conductive patterns must be designed after considering the liquid metal-filled microfluidic channels. For easy injection and flow, the width (f) and height (l) of a microfluidic channel on the top plane were determined to be 0.5 and 0.7 mm, respectively. To realize a ground plane with microfluidic channels, wide meander lines were designed on the bottom substrate. The width (g) and height (m) of a microfluidic channel on the bottom plane were determined to be 2.5 and 1.0 mm, respectively. To design the unit cell of the metamaterial absorber, a finite element method-based ANSYS high-frequency structure simulator was used. In full-wave simulation, an infinite array of a periodic structure was assumed. Therefore, the combination of master and slave boundaries were applied to opposite faces of the solution space. Floquet ports were used to excite electromagnetic waves.

**Figure 2:**
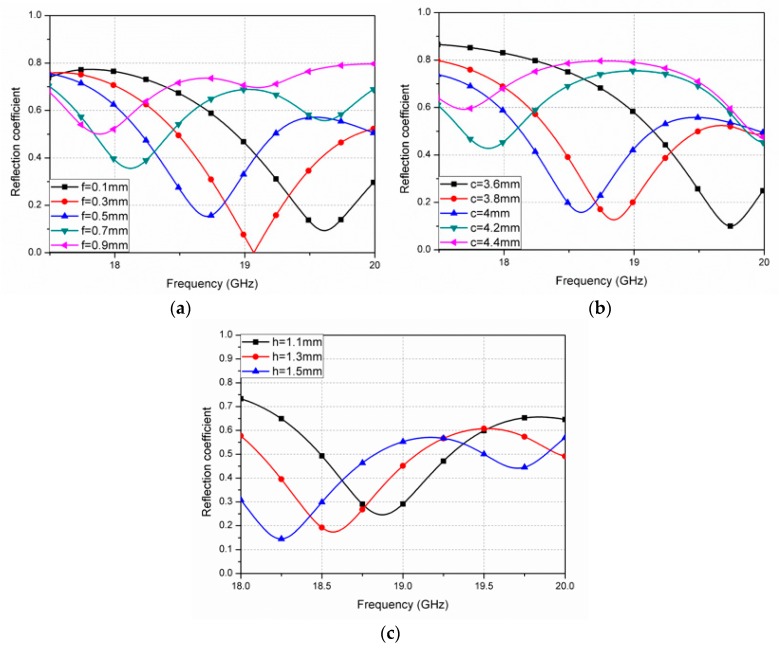
Magnitude of the **s**imulated reflection coefficients (*S*_11_) for the proposed absorber as a function of (**a**) channel width, “f”; (**b**) channel length, “c”; and (**c**) substrate thickness, “h”.

The geometry of the conductive pattern and substrate thickness of the unit cell can be used to determine the reflection coefficient and the resonant frequency of the designed absorber. In Figure 2, the magnitudes of the simulated reflection coefficients are shown for various geometrical parameters of the unit cell. It is observed from Figure 2a that the resonant frequency decreases as the channel width (f) increases. If the channel width is too wide, a lower absorptivity is expected. Figure 2b shows that resonant frequency tends to decrease as the channel length (c) increases. Figure 2c shows the variation in the reflection coefficient as a function of the substrate thickness (h). When “h” increases, both the resonant frequency and the reflection coefficient decrease. Although a thicker substrate results in higher absorptivity, it becomes less stretchable.

**Figure 3:**
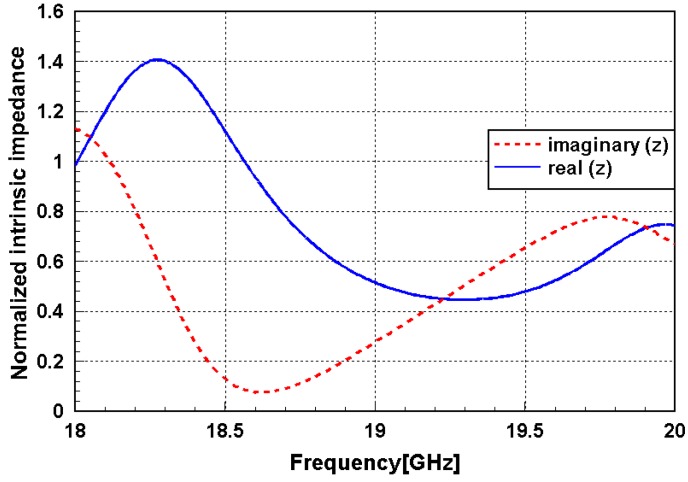
Normalized intrinsic impedance of the proposed metamaterial absorber.

Figure 3 shows the normalized intrinsic impedance of the proposed metamaterial absorber. The complex impedance is normalized to the impedance of free space. The normalized complex impedance of the unit cell can be obtained according to the S-parameter, as follows [27]:

(3) $${\tilde{Z}}_{M}\left(\omega \right)=\sqrt{\frac{{\left(1+S_{11}\right)}^{2}-S_{21}^{2}}{{\left(1-S_{11}\right)}^{2}-S_{21}^{2}}}$$

**Figure 4:**
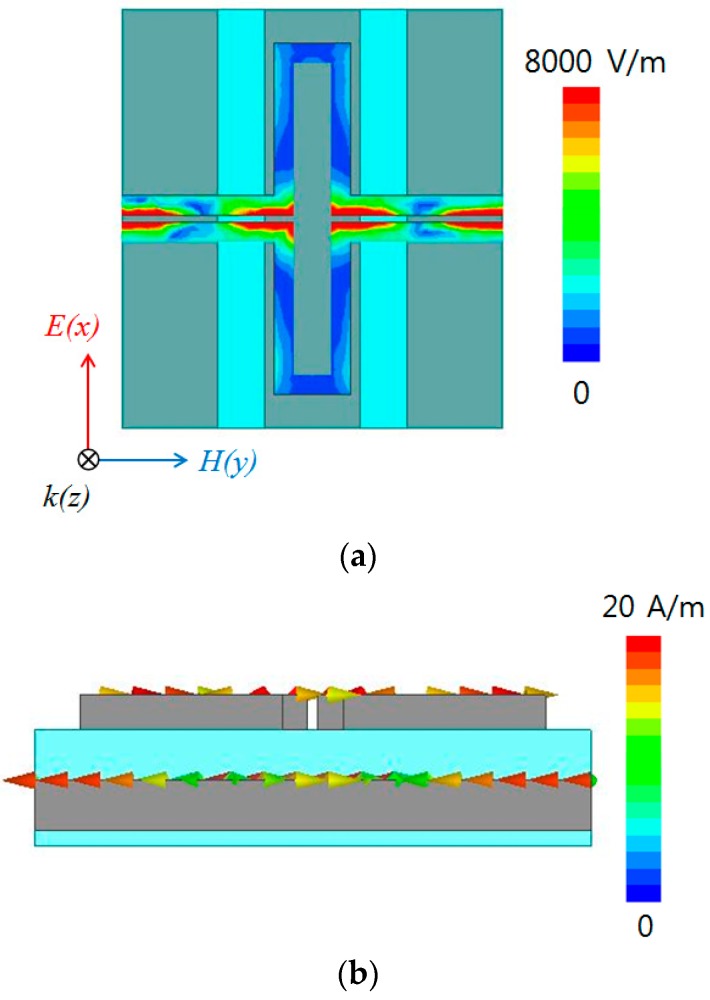
Simulated (**a**) magnitude of electric field distribution and (**b**) vector current density of the designed metamaterial absorber at 18.5 GHz.

The value of the real part is close to unity, and the value of the imaginary part approaches zero at 18.57 GHz. The absorption frequency of the designed absorber is expected to be 18.57 GHz. The absorption phenomenon of the metamaterial absorber is explained by the simultaneous electric and magnetic resonances. The electric field and surface current distributions at 18.5 GHz are given in Figure 4. The electric resonance of the proposed metamaterial was generated from the top and bottom layers of the conductive patterns. As shown in Figure 4a, strong electric coupling was observed at the gaps between the top conductive lines. Figure 4b describes the magnetic resonance, as the vector current densities of the top and bottom layers remained anti-parallel. The anti-parallel currents form a magnetic dipole which functions as a current ring.

## 3. Fabrication

**Figure 5:**
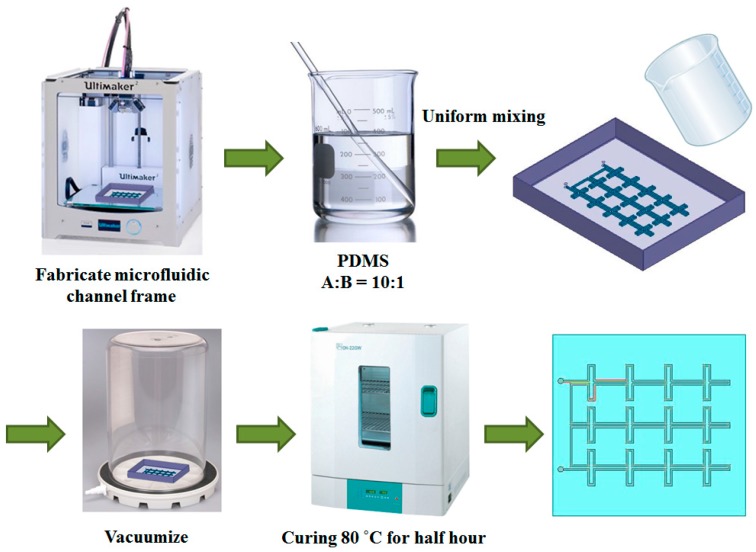
Fabrication process for the designed metamaterial absorber on PDMS.

**Figure 6:**
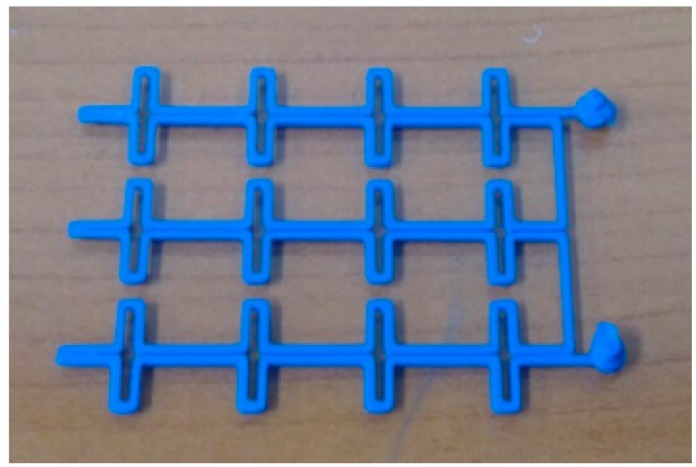
Microfluidic-channel frame fabricated using a 3D printer.

Figure 5 shows the fabrication process for the designed metamaterial absorber. First, microfluidic-channel frames were built. Recent studies have shown that building microfluidic channels using a three-dimensional (3D) printer [28,29] and laser ablation [30] are practical. In this work, a 3D printer is preferred to fabricate the microfluidic-channel frame, in order to avoid the complications, high costs, and large time requirements of conventional methods such as spin-coating and ultraviolet exposure [31,32]. Figure 6 shows the 3D-printed microfluidic-channel frame for the top conductive patterns. Next, a silicone elastomer base and curing agent were mixed at a ratio of 10:1 until thoroughly combined. The mixed PDMS solution was placed in a vacuum chamber to remove air bubbles. After the removal of the air bubbles, the PDMS was baked in an oven at 80 °C for 30 min. Before the solution was baked, it was placed in the microfluidic-channel frame. One side of the PDMS was flat, and the other was patterned with the microfluidic channel. To fabricate the proposed metamaterial absorber, the two PDMS layers with different microfluidic channels and one PDMS layer with no microfluidic channel were used. The PDMS-only middle layer prevents the leakage of the liquid metal. After the PDMS precursor solution is poured onto the microfluidic-channel frame, no holes should remain on the surface of the frame. The holes were eliminated in the microfluidic-channel frame by using ethanol. This process also smoothens the surface of the frame upon its separation from the PDMS.

**Figure 7:**
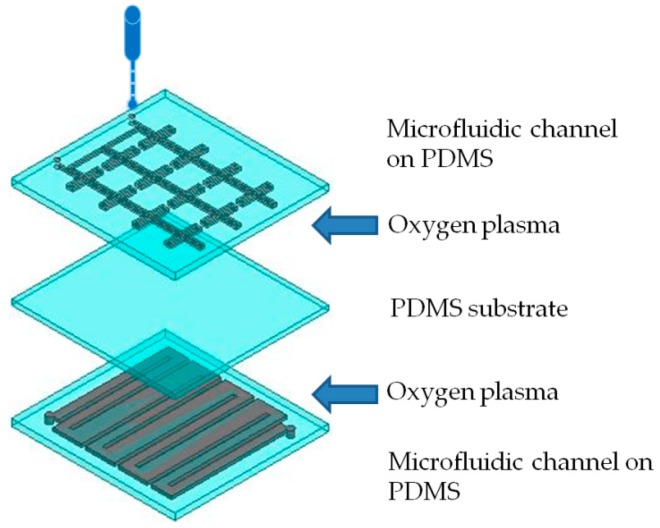
Illustration of the bonding of three PDMS layers.

When three PDMS layers are ready, they must be bonded to each other. Because PDMS has a significant release force, adhesives such as tape are insufficient for adherence. The adhesion of the PDMS layers was increased by using oxygen plasma. The polymer layers were bonded by the attraction caused by the dispersion of ions in the PDMS surface by the oxygen plasma [33]. After plasma treatment of the microfluidic channel-patterned PDMS, the layers were bonded together, as shown in Figure 7.

**Figure 8:**
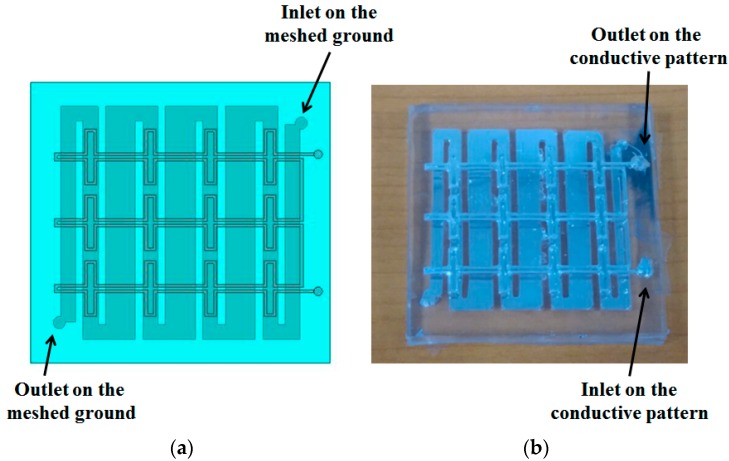
(**a**) Layout of the proposed metamaterial absorber; (**b**) photograph of the fabricated absorber.

As the last step, EGaIn liquid metal was injected into the microfluidic channels on the top and bottom PDMS layers using a syringe with a hose. One inlet hole and one outlet hole were located in each PDMS layer. Figure 8 shows a prototype of the fabricated metamaterial absorber with 4 × 3 unit cells. Its overall physical size is 5.2 cm × 4.4 cm.

## 4. Simulation and Measurement Results

**Figure 9:**
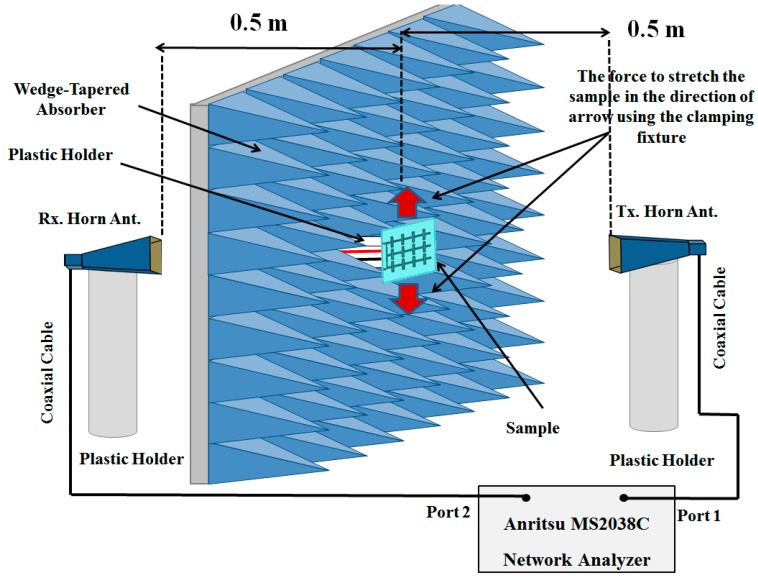
Far-field test environment for measuring the absorptivity of the metamaterial absorber.

Figure 9 shows the far-field test environment for measuring the absorptivity of the fabricated metamaterial absorber. The wedge-tapered absorbing materials were positioned to block the reflected EM wave. Two horn antennas were used as transmitting and receiving antennas. To avoid reflection from the ground floor, the horn antennas were placed on tall plastic holders. An Anritsu MS2038C network analyzer was used to measure the S-parameters. The absorptivity was calculated using *S*_11_ and *S*_21_, as follows:

(4) $$A(\omega )=1-{\left|S_{11}\right|}^{2}-{\left|S_{21}\right|}^{2}$$

We used the time-gating method of the network analyzer to select only the EM waves reflected from the absorber prototype. The distance between the absorber prototype and the horn antenna was fixed at 0.5 m to satisfy the far-field condition, which is given by

(5) $$R>\frac{2D^{2}}{\lambda }$$

where *λ* is the wavelength and *D* is the maximum linear dimension of the antenna. Because 0.5 m is greater than the calculated *R* value of 0.4 m from Equation (5), the distance of 0.5 m satisfies the far-field condition. The reflection coefficient is calibrated as *Г* = −1 with a metal plate.

**Figure 10:**
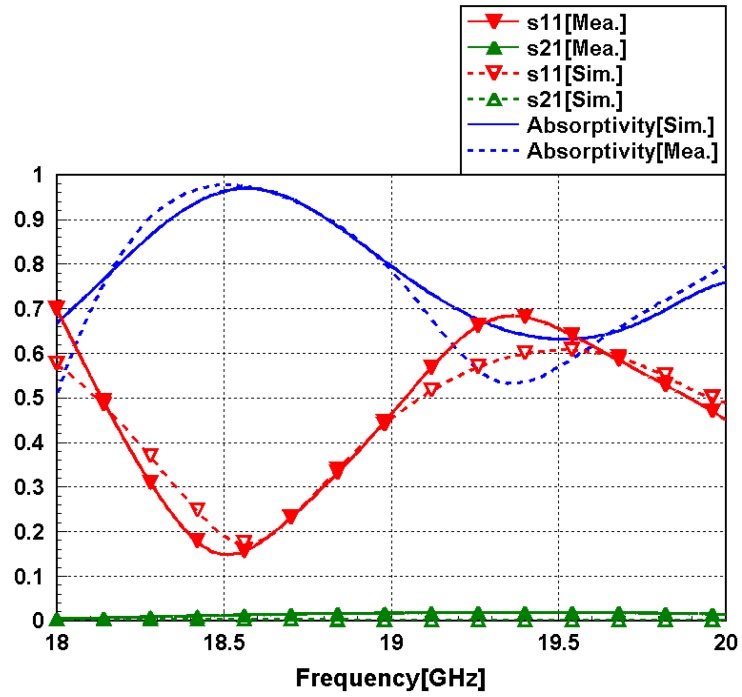
Simulated and measured reflection coefficient (*S*_11_), transmission coefficient (*S*_21_), and absorptivity (A) of the proposed metamaterial absorber.

Figure 10 shows the simulated and measured absorptivity of the proposed metamaterial absorber. The measured absorption frequency was 18.5 GHz, whereas the simulated absorption frequency was 18.6 GHz. The measured absorptivity was 97.8% at 18.5 GHz. Although the ground plane was realized using meander lines, a transmission coefficient of zero was achieved. Good agreement is observed between the simulation and measurement results, as shown in Figure 10. The slight difference is due to manufacturing errors.

**Figure 11:**
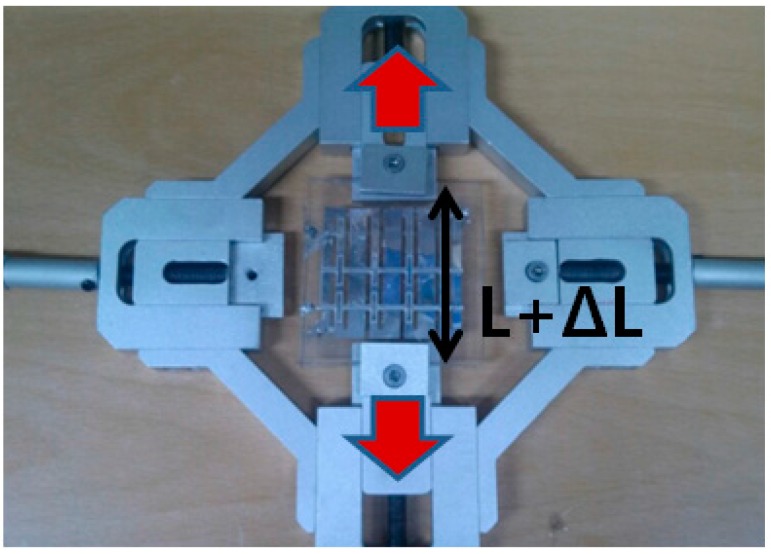
Picture of the proposed metamaterial absorber with a clamping fixture to stretch the absorber.

**Figure 12:**
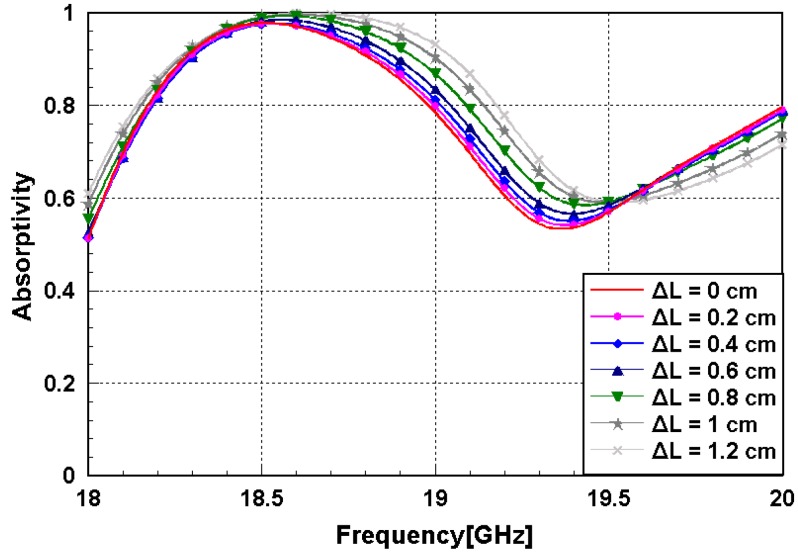
Measured absorptivity of the stretchable metamaterial absorber.

**Figure 13:**
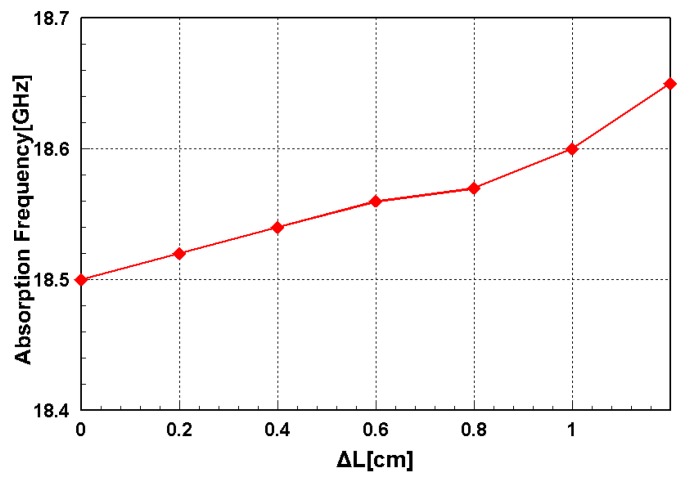
Relationship between the absorption frequency and ΔL.

Because the proposed metamaterial absorber is stretchable, its absorption frequency is measured under different strains. Figure 11 shows the fabricated metamaterial absorber with a clamping fixture that can stretch the absorber. When its absorptivity was measured under the test setup, shown in Figure 8, the metal clamping fixture was wrapped with ferrite-absorbing materials to eliminate reflections from the metal. The absorber prototype was stretched in the directions of the arrows as shown in Figure 10. The prototype had an initial length (L) of 5.2 cm and was stretched to 6.4 cm. Figure 12 shows the measured absorptivity at different values of ΔL. A slight frequency change was observed, while the high absorptivity was maintained. The relationship between the absorption frequency and ΔL is depicted in Figure 13. The value of ΔL is varied from 0 to 1.2 cm in increments of 0.2 cm. The resonant frequency of the stretchable absorber was increased from 18.5 to 18.65 GHz as the absorber was stretched from 0 to 1.2 cm. After the strain was released, the resonant frequency was recovered from 18.65 to 18.5 GHz. This reversible tuning demonstrated the robustness of the proposed stretchable absorber.

The performances of the proposed absorber are compared with those of other tunable absorbers. Table 1 shows a specific comparison with tunable metamaterial absorbers using microelectromechanical systems (MEMS) switches [34], liquid crystal [35], and varactor diodes [36] in terms of peak absorptivity, 80% absorption bandwidth (BW), and tuning ratio (TR). Tuning ratio is defined by

(6) $$TR=100\times \frac{f_{H}-f_{L}}{f_{c}}\;\left(\%\right)$$

where *f*_H_ and *f*_L_ are the highest and lowest frequencies, respectively; *f*_c_ is a center frequency of *f*_H_ and *f*_L_. Compared with other electronically tunable absorbers, the proposed stretchable absorber shows a lower TR. Nevertheless, the proposed stretchable metamaterial absorber has a potential use as a wireless strain sensor. For instance, when a wideband interrogation signal is wirelessly transmitted towards the proposed absorber, an absorption frequency can be measured from the reflected frequency response [37]. Therefore, the strain-level variation can be wirelessly monitored using the variation in absorption frequency.

According to the aforementioned results, the proposed metamaterial absorber can be used as a wireless sensor. With the frequency of the reflected signal from the wireless sensor, we can determine the stretching of the sensor. The proposed metamaterial absorber has several advantages as a sensor. Such a sensor can be used wirelessly, with no battery. Because the proposed absorber is originally an infinite periodic structure, it can be modified for large-area applications. The proposed absorber can be used in various fields, e.g., for measuring the strain of cracked sections in construction materials. The stretched length can be determined based on the frequency of the reflected signal from the proposed absorber.

## 5. Conclusions

A stretchable metamaterial absorber using liquid metal and PDMS is proposed. To achieve the stretchability, we introduced elastomeric PDMS materials and microfluidic channels for injecting liquid metal. The metamaterial unit cell was designed after considering the microfluidic channels. Because a 3D printer was used to fabricate the microfluidic-channel frames, the fabrication time and complexity were reduced. The measured absorptivity of the fabricated absorber was 97.8% at 18.5 GHz. The absorption frequency increased from 18.5 to 18.65 GHz as the absorber was stretched from its initial length to ΔL = 1.2 cm. Therefore, the proposed absorber can be used in wireless strain-sensor applications. In future work, its sensitivity and strain level can be further increased by using thinner substrates and a narrower bandwidth.

## Figures and Tables

**Table 1:** Comparison of the proposed absorber’s performances with other tunable absorbers.

Literature	Tuning Technology	Peak Absorptivity	80% Absorption BW	TR
[34]	MEMS switch	98%	0.1 THz (2.7%)	8.1%
[35]	Liquid crystal	93%	0.26 THz (7%)	7.4%
[36]	Varactor diode	99%	0.1 GHz (0.9%)	2.2%
Proposed work	Stretchable	98%	0.8 GHz (4.3%)	0.8%
